# Diuretic and antioxidant activities of the aqueous extract of leaves of *Vepris heterophylla* (Engl.) R. Let (Rutaceae) in rats

**DOI:** 10.1186/s12906-016-1439-8

**Published:** 2016-12-13

**Authors:** Fidèle Ntchapda, Christian Bonabe, David Romain Kemeta Azambou, Emmanuel Talla, Théophile Dimo

**Affiliations:** 1 Department of Biological Sciences, Faculty of Sciences, University of Ngaoundéré, P.O. Box 454, Ngaoundéré, Cameroon; 2 Department of Chemistry, Faculty of Science, University of Ngaoundéré, P.O. Box 454, Ngaoundéré, Cameroon; 3 Department of Animal Biology and Physiology, Faculty of Science, University of Yaoundé 1, P.O. Box 812, Yaoundé, Cameroon

**Keywords:** *Vepris heterophylla*, Diuretic, Antioxidant, Urine, kidney function, Electrolytes

## Abstract

**Background:**

*Vepris heterophylla* (Rutaceae) is a medicinal plant used empirically in African traditional medicine for many clinical conditions including edematous disorders and hypertension. *V. heterophylla* aqueous extract has been used in northern part of Cameroon by traditional healers for the treatment of arterial hypertension. The study aim was to assess the putative diuretic and antioxidant properties of *V. heterophylla* leaves aqueous extract.

**Methods:**

Adult rats were administered with *V. heterophylla* leaves aqueous extract acutely (24 h) at doses 50, 100, 150, 200 and 250 mg/kg (*per os*). The two positive control groups received the diuretic drugs furosemide (5 mg/kg) and hydrochlorothiazide (HCTZ, 10 mg/kg), while negative control group received only an equivalent volume of distilled water. Urinary elimination of electrolytes in response to treatments was evaluated, together with changes in concentrations of creatinine, urea, aldosterone, glucose and albumin in urine and plasma. Various urinary indicators of kidney function and plasmatic markers of oxidative stress were also assessed.

**Results:**

The findings indicated that the aqueous extract of *V. heterophylla* at doses ranging from 150 to 250 mg/kg caused a significant and dose-dependent increase of urinary water and electrolytes excretion in normal rats. The aqueous extract of the leaves of *V. heterophylla* accelerated the elimination of overloaded fluid. At the maximum of diuretic response, urinary osmolarity decreased significantly when compared with controls. Oral administration of aqueous extract at different doses produced a significant diuresis and slight increase in electrolytes (Na^+^, K^+^ and Cl^−^) excretion. The results obtained were compared with standard drug-furosemide (5 mg/kg) and hydrochlorothiazide (10 mg/kg). These effects were observed predominantly at 250 mg/kg dose.

**Conclusions:**

Our findings strongly suggest that *V. heterophylla* aqueous extract has diuretic and antioxidant activities, and deserves further studies considering the potential for the treatment of hypertension.

## Background

Arterial hypertension is among the most frequent pathologies in elderly worldwide, with an incidence ranging from 40 % (about 65 years patients) to 90 % (patients older than 85) in developed countries [1, 2]. This pathology raises more concerns as it constitutes a major risk of cardiovascular accident. In many of the developing countries, the use of plant drugs against arterial hypertension is increasing because modern life saving drugs are beyond the reach of their countries although they spend 40–50 % of their total wealth on drugs and healthcare [3]. Many investigators demonstrated that studies of herbal plant used in traditional medicine as diuretics were in progressive elevation in the last decades, and might be a precious tool used in human pathology treatment [4]. In Cameroon, different types of complementary and alternative treatments are believed to be effective for treating high blood pressure. Using a diuretic to prevent cardiovascular complications plays an important role in the management of arterial hypertension [5]. These diuretics increase the drainage of water and salt (sodium) into the urine, causing a reduction of blood pressure, a decrease in blood volume, and thus lowering resistance to the flow of blood [6]. Efforts of Cameroonians scientists and traditional therapists are growing in the direction of improvement and enhancement of the use of medicinal plants, to elucidate the pharmacological properties of these plants and possibly to extract active ingredients. The medicinal value of these plants lies in some chemical active substances that produce a definite physiological action on the human body. The most important of these chemically active (bioactive) constituents of plants are: saponin, glycosides and flavonoid compounds. Many of these indigenous medicinal plants are also used for medicinal purposes [7].

*Vepris heterophylla* is a medicinal plant used empirically in the mountainous massifs in the northern part of Cameroon for the treatment of various illnesses such as malaria and cardio-vascular disorders [8]. The vernacular names of this species are Kounikoutchoum (Guiziga, Mofou), Hohoum (Zoulgo), Gougouvetche (Mafa), Kotokolhi (fulfulde) which testifies its importance in this region [9]. The medicinal virtues of this Rutaceae on high blood pressure have already been pointed out by several authors: [10, 11]. The efficacy of *V. heterophylla* leaf extract in bringing relieve to patients with cardiovascular diseases was previously demonstrated. The vascular effects of *V. heterophylla* methanol leaf extract in experimental rat thoracic aortic strips, with a view of providing a pharmacological justification (or otherwise) to the ethno-medical uses of the plant leaf in the management, control and/or treatment of hypertension and certain cardiac dysfunctions was demonstrated [12]. This study indicated that, the vasorelaxation induced by the methanol leaf extract plant was endothelium dependent effect, likely via the NO–cGMP pathway or independent associated mediators such as prostacyclin [13]. A flavonoid (6,8-dihydroxy-4′- methoxyflavone) isolated from stem bark was found to induced nitric oxide-dependent vasodilation in rat aorta [14]. In the same order a triterpen (3β-16β, 23, 29-tetrahydroxyoleane-12-ene) isolated from stem bark was found to increase intracellular Ca^2+^ in rat aorta endothelial cells [15]. To our knowledge, there have been no reports on the diuretic and antioxidant activities of *V. heterophylla* leaves. The present work aim was to measure the diuretic and antioxidant activities of the aqueous extract of *V. heterophylla* leaves in rats.

## Methods

### Animals and procedures

Wistar rats (168.8 ± 2.7 g) of both sexes obtained from Yaoundé’s Pasteur Institute (Cameroon) were reared in the Department of Biological Sciences, Faculty of Sciences (University of Ngaoundéré, Cameroon). Animals were housed under controlled temperature (24 ± 2 °C) and relative humidity (45 ± 10 %), and had *ad libitum* access to food [pellets from Cameroonian National Veterinary laboratory (LANAVET)] and tap water. Animal health status and housing conditions were monitored by a veterinary physician. Preliminary tests were performed as previously described [16]. Briefly, rats received distilled water *per os* (10 mL/kg body weight), and placed individually in metabolic cages. After 6 h, urine was collected and the volume measured. Animals excreting at least 40 % of the volume of distilled water received were selected for the study, and conversely, those excreting less than 40 % were excluded. Then, selected animals were placed individually in metabolic cages, and allowed 7 days for acclimation. Eight experimental groups were obtained by treating *n* = 5 rats (per group) with a specific solution, i.e.: vehicle solution (distilled water, *per os*) for the negative control group, one of the 5 different doses of extract investigated for the 5 test groups (*per os*), and the diuretic drugs furosemide (5 mg/kg) or hydrochlorothiazide (HCTZ) (10 mg/kg) for the positive control groups. Animals were sacrificed by decapitation at the end of the experiment. Arteriovenous blood was collected in heparinized tubes and centrifuged (3000 rev/min for 10 min). The plasma collected was stored at −20 °C for biochemical analyses. The liver and kidneys were dissected out, cleaned of fat material, weighed and stored at −20 °C for biochemical analyses. Urine was collected and the volume determined each hour from the treatment for 6 h (i.e. 1, 2, 3, 4, 5 and 6 h after treatment) and 24 h after in all experimental groups. Electrolyte concentrations (Na+, K+ and Cl^−^) were measured in 24 h urine and in blood plasma obtained from animals sacrificed 24 h after treatment. Experimental procedures were approved by the institutional Animal Care and Use Committee and the research was approved by the Ethics Committee of the Department of Biological Science of the ﻿University of Ngaoundéré (ECDBSUN 15/01/2015/UN/FS/DSB).

### Concentrations of other blood molecules

A two-way digital spectrophotometer (Secomam RS232C, Secomam SAS, France) was used to determine the concentrations of urea, glucose, albumin and creatinine in plasma and urine samples. Similarly malondialdehyde concentration was determined in plasma, and catalase, Glutathion, hydroperoxide and protein concentrations were determined in hemolysates of blood pellets and in liver homogenates. Aldosterone concentration in the plasma was measured using radioimmunoassay (assay kit Aldo RIACT, ALPCO Diagnostics, USA).

### Plant extract preparation

#### Plant material collection

*Vepris heterophylla* leaves were harvested in August 2013 in Kaliyao locality of Mokolo (Far North Region, Cameroon) (10 ° 39.214 ′N, 14 ° 24.145′ E, 375 m asl). The identification and authentication were done in the National Herbarium of Cameroon where a voucher specimen was kept under the No 61615/HNC.

#### Aqueous extract leaves processing

Fresh leaves of *V. heterophylla* were soaked in distilled water (1000 g for 1 L at room temperature) for 12 h. The mixture was shaken vigorously for 10–15 s and allowed to stand for about 30 min and then filtered through a 150 μm aperture sieve to obtain the macerate. The macerate was filtered through Whatman filter paper N^o^ 3, and the aqueous extract was there after lyophilized to give a yield of 10.26 %. The sample was then placed in air-tight containers and refrigerated at −20 °C until use.

#### Aqueous extract doses

The doses were given starting from the dose of the traditional expert. The solution of *V. heterophylla* extract with the highest concentration tested was prepared by dissolving 500 mg of the concentrated crude extract obtained previously in 10 ml of distilled water (50 mg/mL concentration). The other solutions used in the study were 4:5, 3:5, 2:5 and 1:5 dilutions of this solution in distilled water. Solutions were given *per os* in a volume of 5 ml/kg body weight, thus, the increasing doses of aqueous extract of *V. heterophylla* tested were 50, 100, 150, 200 and 250 mg/kg.

#### Determination of urinary and/or plasma concentrations

Osmolar clearance (Cosm) was calculated using plasma osmolality (POSM), urinary osmolarity (Uosm) and urine flow (V) according to the following formula: Cosm = Uosm x V/POSM. Urinary and plasma concentrations of Na^+^, K^+^ and Cl^−^ ions were evaluated using flame photometry (Jenway PFP 7, Bibby Scientific, USA), following standard protocols. Osmolarity of plasma and urine samples were measured by cytometry using an osmometer (Knauer). Urinary natriuresis was measured during the diuretic response, particularly at the maximum excretion rate. Doses of Na^+^ and K^+^ were calculated as indicators of saluretic activity and the ratio Na^+^/K^+^ was calculated for the natriuretic activity. And to estimate the carbonic anhydrase inhibition activity, the ratio of Cl^−^ ions to Na^+^ and K^+^ ions was calculated [17]. A two-way digital spectrophotometer (Secomam RS232C, Secomam SAS, France) was used to determine the concentrations of urea, glucose, albumin and creatinine in plasma and urine samples. Similarly malondialdehyde concentration was determined in plasma, and catalase, hydroperoxide and protein concentrations were determined in hemolysates of blood pellets and in liver homogenates. Aldosterone concentration in the plasma was measured using radioimmunoassay (assay kit Aldo RIACT, ALPCO Diagnostics, USA).

#### Phytochemical studies

In order to identify the chemical structure of the compounds responsible for the diuretic activity, preliminary tests of the phytochemical study were conducted following the procedures described by Trease and Evans [18]. Briefly, Essential oils from the aqueous extract of *V. heterophylla* were extracted with hexane. These extracts were then stitched onto plates of thin layer chromatography on silica, the first disclosure was obtained by ultraviolet radiation (254 nm and 365 nm) and then with vanillin. Analytical tests for the identification of different families of metabolites in crude extracts of the leaves were performed at the national Institute of Medicinal Plants for Medicinal research (IMPM, Cameroon).

#### Statistical analyses

Data from test groups and positive control groups were compared to negative control group using one-way ANOVA followed by LSD test for post hoc analysis, using Origin software (OriginLab, Northampton, MA, USA). Changes with P values lower than 0.05 were considered significant. Data are presented as mean ± SEM.

## Results

### Phytochemical study

Phytochemical screening performed on crude extracts revealed the presence of several primary and secondary metabolites such as fatty acids, Athraquinones, volatile oils, glycosides, saponins, tannins, coumarins and triterpenes. Phenolic compounds and sterols were also present in the extract. The presence of flavonoids and alkaloids was significant. The various phytochemical compounds detected are known to have beneficial importance in industrial and medicinal sciences. These initial results suggest that the aqueous extract of leaves of *V. heterophylla* contains several chemical compounds whose potential biological activity remains to be demonstrated.

### Overload eliminated after 1h and latency to first urination

**Fig. 1:**
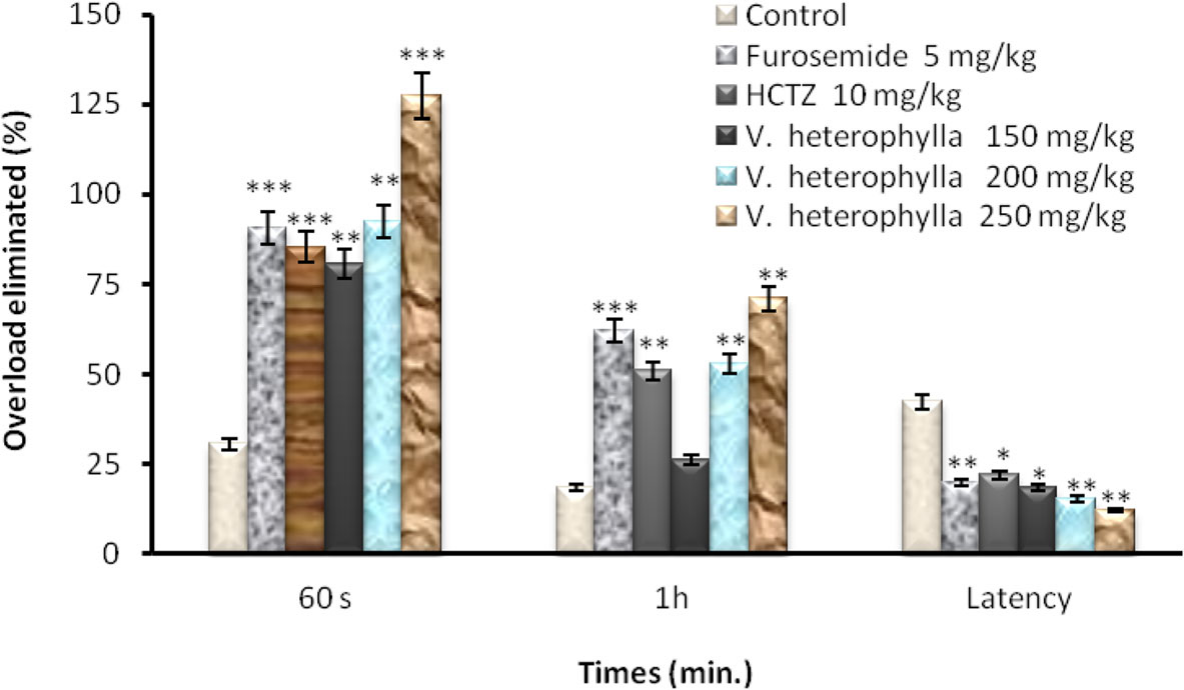
Overload eliminated and urination latency. Overload eliminated after 1 h and latency to first urination by rats treated with the three highest doses of *V. heterophylla* aqueous extract tested, administered with an equivalent volume of distilled water (negative controls), or rats treated with one of the two diuretic drugs used as positive controls. Note that all treatments induced at least a 3-fold increase in the overload elimination compared with negative controls. ANOVA + LSD test against negative control group: *P < 0.05, **P < 0.01, ***P < 0.001

All treatments induced at least a 3-fold increase in the overload elimination compared with negative control group after 60s. Only 30.54 % overload was eliminated in the negative control group, whereas HCTZ eliminated 85.34 % and furosemide 90.66 % (*P* < 0.001). The response of the extract was dose-dependent, and eliminated 80.62 % of overload at 150 mg/kg (*P* < 0.001 against negative control group), 92.44 % at 200 mg/kg (*P* < 0.01) and 127.38 % at 250 mg/kg (*P* < 0.001) (Fig. 1). The overload eliminated after 1 h by rats treated with the three highest doses of extract (150, 200 and 250 mg/kg) were respectively 26.23; 52.88 and 70.87 %, whereas HCTZ eliminated 51.04 % furosemide 61.87 % and control 18.31 % (*P* < 0.001) (Fig. 1). The extract displayed a dose-dependent decrease as the first urination latency with 18.38 min (*P* < 0.05), 15.24 min (*P* < 0.01), and 12.11 min (*P* < 0.01) at doses 150, 200 and 250 mg/kg. Negative control group average first urination latency was 42.34 min, furosemide 19.66 min (*P* < 0.01), and HCTZ 21.87 min (*P* < 0.05) (Fig. 1).

### Urinary volume and electrolyte excretion

Table 1 shows the changes in the total volume of urine excreted by rats after 24 h following treatment with various doses of *V. heterophylla* aqueous extract, administration of an equivalent volume of distilled water (negative controls), or treatment with one of the two diuretic drugs used as positive controls (furosemide, HCTZ). The doses 50 mg/kg and 100 mg/kg were not significant (data not shown). The volume of urine went from 231.31 ± 2.38 ml/kg/24 h in control group to 393.04 ± 3.62 ml/kg/24 h in the group treated with the highest dose. A single dose-response administration of the aqueous extract of *V. heterophylla* (150, 200 and 250 mg/kg) significant increased (*P* < 0.05) the volume of urine 24 h later. However, the urinary excretion was dose dependant (Table 1). The volume of urine of 208.22 ± 2.43 ml/kg/24 h in controls (distilled H_2_0) significantly increased to 254.11 ± 3.23 ml/kg/24 h (22.11 % increment) at the dose of 150 mg/kg. At the dose of 200 mg/kg, the volume of urine increased by 37.50 %. The highest dose (250 mg/kg), induced 72.11 % increase of the treated group. All treatments increased the urinary excretion of Na^+^; Cl^−^ and k^+^ compared to negative control group; the extract response was dose-dependent (Table 1).

**Table 1:** Effects of the aqueous extract of *V. heterophylla* on the urinary volume and excretion of Na^+^, Cl^−^ and K^+^

Urinary volume and excretion of Na^+^, Cl- and K^+^					
Drugs (mg/kg)		Urinary volume (mL/kg/24 h)	Na^+^ (mEq/kg/24 h)	Cl^−^ (mEq/kg/24 h)	K^+^ (mEq/kg/24 h)
Control		208.22 ± 2.43	23.32 ± 1.11	19.33 ± 1.41	23.11 ± 0.66
Extract	150	254.11 ± 3.23***	59.22 ± 2.23***	53.16 ± 1.32***	44.18 ± 1.31***
	200	286.13 ± 2.13***	112.33 ± 1.33***	107.33 ± 1.41***	60.34 ± 1.22***
	250	358.08 ± 3.13***	166.28 ± 1.22***	158.67 ± 1.42***	52.33 ± 2.14***
Furosémide (5 mg/kg)		398.12 ± 1.77***	101.66 ± 2.11***	99.77 ± 2.22***	40.77 ± 2.22***
Amiloride H. (10 mg/kg)		237.11 ± 4.33**	81.77 ± 1.31***	77.22 ± 2.11***	29.66 ± 1.13**

Values are means ± S.E.M., *n* = 5, ** < 0.01, *** < 0.001, significant difference compared to the control

After 24 h, the extract significantly (*P* < 0.001) increased the urinary excretion of Na^+^ and Cl^−^ from 23.32 ± 1.11 mEq/kg and 19.33 ± 1.41 mEq/kg (negative control group), respectively, to 59.22 ± 2.23 mEq/kg and 53.16 ± 1.32 mEq/kg (150 mg/kg), 112.33 ± 1.33 mEq/kg and 107.33 ± 1.41 mEq/kg (200 mg/kg), and 166.28 ± 1.22 mEq/kg and 158.67 ± 1.42 mEq/kg (250 mg/kg). The urinary excretion of Na^+^ and Cl^−^ induced by furosemide were 101.66 ± 2.11 mEq/kg and 99.77 ± 2.22 mEq/kg (*P* < 0.001), respectively and HCTZ 81.77 ± 1.31 mEq/kg and 77.22 ± 2.11 mEq/kg (*P* < 0.001) (Table 1). The urinary excretion of K^+^ induced by extract at the dose of 250 mg/kg were 52.33 ± 2.14 mEq/kg and furosemide 40.77 ± 2.22 mEq/kg (*P* < 0.001), HCTZ 29.66 ± 1.13 mEq/kg (*P* < 0.001) (Table 1).

All experimental groups had a significantly higher slope (*P* > 0.01) than the negative control group (*y* = 0.15x + 16.9, R^2^ = 0.59), furosemide (*y* = 11.5x + 85.9, R^2^ = 0.99), HCTZ (*y* = 10.4x + 60.9, R^2^ = 0.98), and extract-treated groups (*y* = 13.1x + 165.6, R^2^ = 0.98). The effects of treatment with extract or diuretic drugs on K^+^ amount excreted was also more marked with time: extract-treated groups (*y* = 5.7x + 23.9, R^2^ = 0.99), furosemide (*y* = 3.6x + 13.2, R^2^ = 0.98) and HCTZ (*y* = 4.5x + 10.1, R^2^ = 0.97).

### Urine output index and pH

**Fig. 2:**
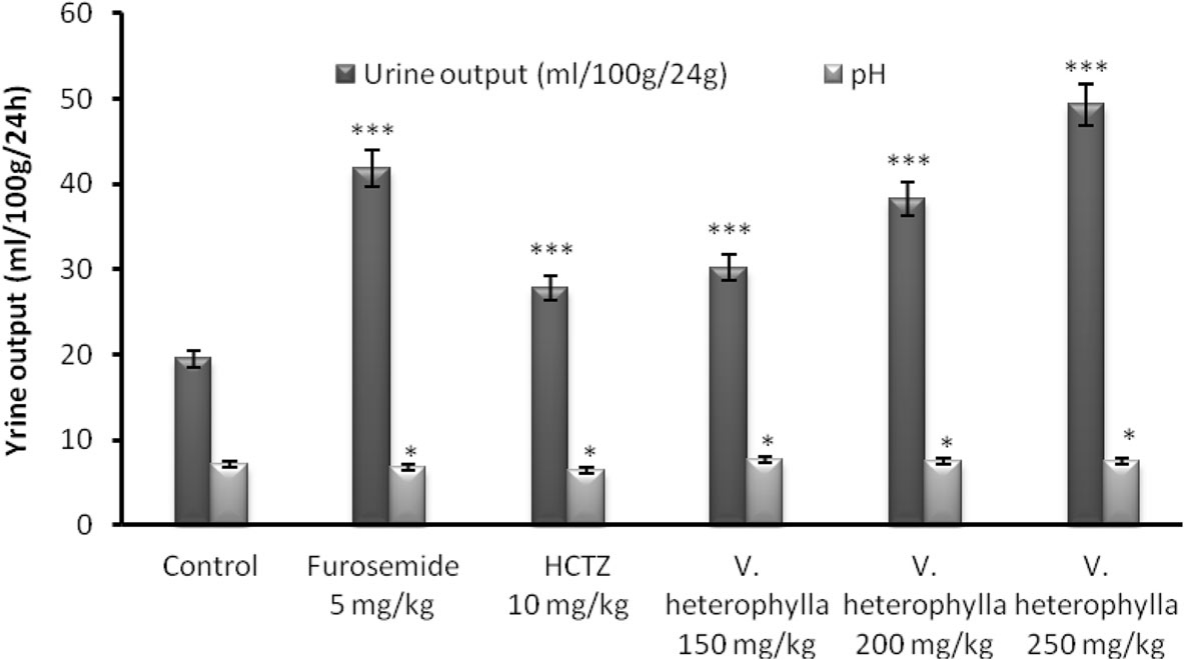
Effects of the aqueous extract of *V. heterophylla* on the urine output and pH. Values are means ± S.E.M., *n* = 5, * < 0,05, *** < 0,001, significant difference compared to the control

The urinary pH in negative control group was slightly decreased in groups treated with furosemide (6.68 ± 0.1) or HCTZ (6.54 ± 0.1), and slightly increased in groups receiving the extract (7.6), with the doses (150, 200 and 250 mg/kg). However, these changes were not statistically significant compared to the control (Fig. 2). Diuretic index of the plant extract is lower than that of furosemide (1.91) and higher than that of HCTZ (1.13) at the dose 150 mg/kg (1.22), 200 mg/kg (1.37) and 250 mg/kg (1.72) (Table 2). At the dose of 150 mg/kg, *V. heterophylla* showed a significantly (*p* < 0.05) high urinary pH when compared to the control group (Fig. 2). The pH values (7.6 ± 0.1) of urine treated with the extract of *V. heterophylla* were higher than the control group (7.1 ± 0.2). Doses of 150 and 200 mg/kg showed a significantly increased pH values. However, the pH value (7.6 ± 0.1) of urine of animals treated with pharmacological substances were lower than that of rats treated with extract at the doses of 150; 200 and 250 mg/kg (7.6 ± 0.1) (Fig. 2).

**Table 2:** Effects of the aqueous extract of *V. heterophylla* on urine output index and electrolytic excretion index in 24 h of urine collection

Drugs (mg/kg)		Diuretic index	Na^+^ index	Cl^−^ index	K^+^ index	*n*
Control		1.00	1.00	1.00	1.00	5
Extract	150	1.22	2.56	2.78	1.91	5
	200	1.37	4.86	5.63	2.60	5
	250	1.72	7.21	8.31	2.26	5
Furosémide (5 mg/kg)		1.91	5.22	5.21	1.73	5
Amiloride H. (10 mg/kg)		1.13	4.39	4.05	1.26	5

*n* number of animals used in each group

diuretic index: urine volume of test group/urine volume of control group

Na^+^ index, sodium excretion in test group/sodium excretion in control group

K^−^ index, potassium excretion in test group/potassium excretion in control group

### Saluretic and natriuretic activities

Saluretic and natriuretic activities were significantly increased by diuretic drugs (*P* < 0.001) and by the extract (*P* < 0.001) at the highest dose tested (Table 3). Similarly, carbonic anhydrase inhibition was also increased by diuretic drugs (*P* < 0.01) and by the extract (*P* < 0.01 at the highest dose tested). On the same hand, carbonic anhydrase inhibition, saluretic, natriuretic, diuretic, as well as Na^+^ and Cl^−^ indexes were high for in all groups, but the extract effect at 250 mg/kg (higher dose used) was 2-fold higher than the diuretic drugs at the dose used (Table 3). Notably, K+ index was only slightly increased by the diuretic drugs and the extract (Table 2).

**Table 3:** Effects of the aqueous extract of *V. heterophylla* on saluretic and natriuretic activity from 24 h of urine

Drugs (mg/kg)		Saluretic (Na^+^ + Cl^−^)	Natriuretic (Na^+^/k^+^)	CAI Cl/(Na + K)	Saluretic index	Natriuretic index	CAI index
Control		42.65 ± 2.52	1.00 ± 1.68	0.45 ± 0.55	1.00	1.00	1.00
Extract	150	112.38 ± 3.55***	1. 34 ± 1.70*	0.47 ± 0.37*	2.63	1.34	1.04
	200	219.66 ± 2.74***	1.86 ± 1.09**	0.48 ± 0.51**	5.15	1.86	1.06
	250	324.95 ± 2.64***	3.17 ± 0.57***	0.48 ± 0.53**	7.61	3.17	1.06
Furosémide (5 mg/kg)		201.43 ± 4.33***	2.49 ± 0.95***	0.49 ± 0.51**	4.72	2.49	1.08
Amiloride H. (10 mg/kg)		158.99 ± 3.42***	2.75 ± 1.15***	0.48 ± 0.61**	3.72	2.75	1.06

Values are means ± S.E.M., *n* = 5, * < 0.05, ** < 0.01, *** < 0.001, significant difference compared to the control

*CAI*, carbonic anhydrase inhibition

saluretic index: saluretic activity in test group/saluretic activity in control group

natriuretic index: natriuretic activity in test group/natriuretic activity in control group

carbonic anhydrase inhibition index, CAI activity in test group/CAI activity in control group

### Urinary indexes of kidney function

Treatments with the various doses of extract and the diuretic drugs decreased the GFR from 1.74 ± 0.12 mL/min in the negative control group to 1.56 ± 0.16 mL/min, (extract at 250 mg/kg, *P* < 0.01) and 1.49 ± 0.22 mL/min (furosemide, *P* < 0.001), 1.46 ± 0.32 mL/min (HCTZ, *P* < 0.001). The analysis of the collected urine of rats 24 h after administration of a single dose of the extract of *V. heterophylla* revealed no trace of glucose and albumin. The effect of *V. heterophylla* aqueous extract and diuretic drug treatment (compared to negative control group) on indexes of kidney function in urine produced in the 24 h following the treatment are shown in Table 4. The aqueous extract of *V. heterophylla* caused no significant change in rate of urinary creatinine (Table 4). Urea concentration in the urine was significant (*p* < 0.05) decreased by 8.33 % at dose 250 mg/kg (Table 4). Osmotic clearance significantly increased by 45.65 % at the highest dose of extract. The creatinine clearance also decreased 35.10 % (Table 4). Osmolar clearance and free water clearance were increased, while urinary osmolarity decreased.

**Table 4:** Effects of the aqueous extract of *V. heterophylla* on index kidney function

Drugs (mg/kg)	Control	150 mg/kg	200 mg/kg	250 mg/kg	Furosemide (5 mg/kg)	HCTZ (10 mg/kg)
Creatinine (mg/24 h)	26,66 ± 1,76	17,77 ± 1,14***	23,45 ± 1,22**	27,54 ± 1,34*	22,33 ± 1,32**	28,67 ± 1,43
CreatC (mL/min)	0,027 ± 0,33	0,025 ± 0,14*	0,023 ± 0,24**	0,021 ± 0,11***	0,022 ± 0,31**	0,020 ± 0,22***
Urea (g/24 h)	26,15 ± 1,16	22,25 ± 1,01***	23,14 ± 1,23*	24,45 ± 1,66	24,76 ± 1,42**	22,68 ± 0,34***
Uosm (mosmol/kg)	198,45 ± 8,73	109,11 ± 12,98***	112,31 ± 13,46***	125,43 ± 11,24***	169,56 ± 12,21***	166,62 ± 8,11***
GFR (mL/min)	1,74 ± 0,12	1,65 ± 0,33*	1,62 ± 0,11*	1,56 ± 0,16**	1,49 ± 0,22***	1,46 ± 0,32***
Cosm (mL/min)	0,046 ± 0,013	0,044 ± 0,021	0,047 ± 0,032	0,049 ± 0,043**	0,078 ± 0,046***	0,067 ± 0,044***
CH20. (mL/min)	0,056 ± 0,012	0,054 ± 0,013	0,056 ± 0,011	0,066 ± 0,016***	0,068 ± 0,015***	0,066 ± 0,018***

Values are means ± S.E.M., *n* = 5, * < 0,05, ** < 0,01, *** < 0,001, significant difference compared to the control

*CreatC* creatinine clearance, *Uosm* urinary osmolarity, *GFR* glomerular filtration rate, *Cosm* Osmolar clearance, *CH*_*2*_ *0* free water clearance

### Serum parameters and oxidative stress markers

Furosemide and HCTZ and the extract at the dose of 250 mg/kg induced significant increases (4.49, 2.68 and 5 % respectively, *p* < 0.05) in albumin level (Table 5). Concentrations of Na^+^ and K^+^ ions were significantly increased (518.18 % and 218.88 % respectively in animals receiving the extract at 250 mg/kg, *p* < 0.05) (Table 5). The extract and diuretic drugs induced significant increases (*p* < 0.05) in serum creatinine and urea levels. Increases in plasma osmolality and aldosterone levels were observed (*P* < 0.001) (Table 5). Table 6 showed the effects of the acute administration of *V. heterophylla* aqueous extract at the dose of 250 mg/kg on markers of oxidative stress. Glutathione concentration in plasma were decreased (*P* < 0.01). The extract also induced a significant decrease in hydroperoxide amount in liver homogenates (*P* < 0.001), and an increase in blood plasma (*P* < 0.05). Catalase activities in liver homogenates and in hemolysates were significantly decreased (*P* < 0.05). Protein amounts were decreased in liver homogenates (*P* < 0.01) and increased in hemolysates (*P* < 0.05). Plasma and liver malondialdehyde amounts were significantly decreased (*p* <0.05).

**Table 5:** Effects of the aqueous extract of *V. heterophylla* on the serum parameters

Drugs (mg/kg)		glucose (mg/dl)	Creatinine (mg/dl)	Urea (mg/dl)	Albumin (g/l)	Aldosterone (pg/mL)	Na^+^ (méq.L–1)	K^+^ (méq.L–1)	POSM (mosmol/kg)
Control		89,22 ± 2,11	0,55 ± 0,22	21,12 ± 0,38	42,52 ± 1,14	279,34 ± 13,41	1,76 ± 0,43	1,80 ± 0,77	269,11 ± 11,11
Extract	150	90,45 ± 2,34*	0,62 ± 0,45*	22,38 ± 1,11	43,66 ± 1,22	281,22 ± 21,55	6,42 ± 0,75***	3,79 ± 1,66***	277,52 ± 12,33**
	200	91,66 ± 4,33*	0,66 ± 0,57**	23,89 ± 2,44**	44,72 ± 2,34**	287,57 ± 12,12**	8,22 ± 1,56***	4,78 ± 2,52***	279,66 ± 21,42***
	250	95,88 ± 3,41*	0,87 ± 0,29***	26,77 ± 2,33***	44,65 ± 3,67***	306,11 ± 32,22***	10,88 ± 1,56***	5,74 ± 1,16***	285,33 ± 23,43***
Furosemide (5 mg/kg)		95,67 ± 4,98*	0,75 ± 0,23***	24,56 ± 3,33**	44,43 ± 1,63**	302,53 ± 23,35***	11,22 ± 1,42***	6,73 ± 1,34***	284,21 ± 24,14***
HCTZ (10 mg/kg)		94,22 ± 3,55*	0,74 ± 0,61***	26,22 ± 1,13***	43,66 ± 1,32***	292,88 ± 11,23***	7,31 ± 1,72***	9,89 ± 2,51***	286,44 ± 31,56***

Values are means ± S.E.M., *n* = 5, * < 0,05, ** < 0,01, *** < 0,001, significant difference compared to the control

*POSM* plasma osmolality

**Table 6:** Effects of the aqueous extract of *V. heterophylla* on markers of oxidative stress

	Homogenate				plasma			haemolysates	
Treatments	MDA (μM/100 g of tissue)	ROOH (μM/100 g of tissue)	CAT (mMH2O2/min/g of protein)	Protein (g/100 g of tissue)	Glutathion (mmol/L)	MDA (μM/l)	ROOH (μM/l)	CAT (mMH2O2/min/g of protein)	Protein (g/l)
Control 250 mg/kg	10.45 ± 1.66	1.66 ± 0.11	0.03 ± 0.02	58.11 ± 0.43	0.45 ± 0,021	19.77 ± 1.70	0.03 ± 0.01	0.01 ± 0.00	39.79 ± 4.21
	8.89 ± 0.77*	0.88 ± 0.34***	0.16 ± 0.03*	36.67 ± 3.23**	0.26 ± 0,033**	8.45 ± 2.11***	0.05 ± 0.01*	0.12 ± 0.04***	49.98 ± 0.34*

Values are means ± S.E.M., *n* = 5, *P < 0.05, *P < 0.01, *P < 0.001, significant difference compared to control rat

*MDA* malondialdehyde, *ROOH* 'hydroperoxyde, *CAT* catalase

## Discussion

Preliminary phytochemical studies showed that aqueous extract of the leaves of *V. heterophylla* contains several chemical compounds that could be partially or fully responsible for the increase of diuresis and moderate natriuretic activity. Saponin is used as a mild detergent and in intracellular histochemistry staining to allow antibody access to intracellular proteins. In medicine, it is used in hypercholesterolaemia, hyperglycaemia, diuresis, antioxidant, anti-cancer, anti-inflammatory and weight loss etc. It is also known to have anti-fugal properties [19]. Tannin are reported to exhibit antiviral, antibacterial, anti-tumor activities. It was also reported that certain tannin are able to inhibit HIV replication selectivity and is also used as diuretic [20]. Cardiac glycosides are known to work by inhibiting the Na^+^/K^+^ pump. This causes an increase in the level of sodium ions in the myocytes, which then lead to a rise in the level of calcium ions. This inhibition increases the amount of Ca^2+^ ions available for contraction of the heart muscle, which improves cardiac output and reduces distention of the heart; thus, they are used in the treatment of congestive heart failure and cardiac arrhythmia. They are also, used to strengthen a weakened heart and allow it to function more efficiently, though the dosage must be controlled carefully, since the therapeutic dose is close to the toxic dose [21]. Plant steroids are known to be important for their cardiotonic activities, they possess insecticidal and anti-microbial properties. They are also used innutrition, herbal medicine and cosmetics, they are routinely used in medicine because of their profound biological activities [21]. Flavonoid have been referred to as nature’s biological response modifiers because of strong experimental evidence of their inherent ability to modify the body’s reaction to allergies, virus and carcinogens. They also show diuresis, anti-allergic, anti-inflammatory, anti-microbial and anti-cancer activity [19].

Findings of the study also indicated that *V. heterophylla* may have antioxidant effects. Comparable observations were reported in a number of other plants with diuretic properties [22]. In addition, the extract also decreased hydroperoxide levels in homogenates, malondialdehyde levels in plasma and the activity of catalase in homogenates and hemolysates, which are markers of oxidative stress [23]. The present study indicated that the aqueous extract of *V. heterophylla* at doses ranging from 150 to 250 mg/kg caused a significant and dose-dependent increase of urinary water and electrolytes excretion in normal rats. It was noted that *V. heterophylla* treatment caused increase in both water and electrolytes excretion qualitatively similar to furosemide which is known by its potential saluretic and diuretic effects [24]. Determination of urinary carbonic anhydrase inhibition revealed that carbonic anhydrase inhibition was not significantly increased. This shows that diuretic activity of aqueous extract of *V. heterophylla* does not make use of carbonic anhydrase inhibition as its mechanism of action [25]. The increase in the ratio of concentration of excreted sodium and potassium ion indicates that the extract increases sodium ion excretion to a greater extent than potassium which is a very essential quality of a good diuretic with lesser hyperkalemia side effect [26].

This study suggest that the aqueous extract leaves of *V. heterophylla* administrated *per os* had stronger diuretic effects at the doses used in acute studies. The urinary output of rats after oral administration of aqueous extract of *V. heterophylla* showed a statistical difference observed between the test groups given the extract and the control groups during the 1st h and after 24 h. There is biological significance in urine volume of positive control in relation to the negative control during the 1st h and at the 24th h. Similar observations were reported in studies assessing the other plants with diuretic activity such as *Retama raetam* [27], and *Ficus glumosa* [17]. The acute administration of the extract at the dose with the more marked response (250 mg/kg) induced an increase of 82.58 % in urinary excretion (compared to negative control group), against 91.20 % and 13.87 % with furosemide and HCTZ, respectively. Diuretics modulate the volume and composition of body fluids in variety of clinical conditions like hypertension. The extract also accelerated the elimination of fluid overload and decreased the latency of the first urination 30.54 % overload was eliminated in the negative control group, whereas HCTZ eliminated 85.34 % and furosemide 90.66 %. The response of the extract was dose-dependent, and eliminated 80.62 % of overload at 150 mg/kg, 92.44 % at 200 mg/kg and 127.38 % at 250 mg/kg against negative control group. and the diuretic index of groups treated with the extract was higher (1.72 at 250 mg/kg) than HCTZ -treated (1.13) but lower than furosemide-treated (1.91). Such rapid diuretic activity may be due to very high concentration of active molecules of the saponin, Cardiac glycosides and flavonoïd families [28], which presence in extracts of *V. heterophylla* was detected by phytochemical analysis in our study and previously reported [13]. The increase natriuresis in response to acute treatment of aqueous extract of leaves of *V. heterophylla* may partly explain the increase in diuresis [29, 30]. *V. heterophylla* also caused the acidification of urine. There was a significant reduction in the osmolarity of urine in rats treated with the extract. *V. heterophylla* may impair the basal secretion of ADH and reduce the responsiveness of uriniferous tubules to the action of ADH. Inhibition of ADH causes polyurea with low osmolarity [31]. Aldosterone hormone measured by radioimmunoassay was slightly increased in animals treated with aqueous extract, and the lack of correlation between plasma aldosterone and sodium concentration in the blood as well as in urine seem to imply that aldosterone is not involved in the natriuresis observed and suggested that stimulation of diuresis by the aqueous extract of the leaves of *V. heterophylla* could be similar to that of furosemide. furosemide increases urinary excretion of sodium by inhibiting Na^+^/K^+^/2Cl^−^ symporter (co-transporter system) in the thick ascending limb of the Henley loop [32], while HCTZ inhibits the Na^+^/Cl^−^ symporter (co-transporter system) in the distal convoluted tubule, by competing for the Cl^−^ binding site, and increasing the excretion of Na^+^ and Cl^−^ [33]. Whether the extract induces the suppression of renal tubular reabsorption of water and electrolytes by one of these processes or by another mechanism is still to be determined. The increase of the Na^+^ excretion tend to reduced GFR by increasing the Na^+^ load available for Na^+^/K^+^ exchange, stimulating further such exchange by hyperaldosteronism, which causes a reduction in blood volume [34].

Glucose and albumin were not present in treated rats’ urine, and no significant change was observed in the urinary creatinine levels. Instead, a marked reduction was observed in the concentration of urea in the urine compared to negative control group, the K^+^ plasmatic concentration was increased, and Na^+^ and Cl^−^ concentrations in the plasma were significantly decreased. Glomerular filtration measured by creatinine clearance does not vary according to treatment compared to controls, which suggest that the increase in diuresis would rather have a tubular origin as seems to show the clearance of free water [16].

Taken together, these results indicate that *V. heterophylla* may act as a loop diuretic which inhibit the Na^+^/K^+^/Cl^−^ co-transporter system in the thick ascending loop of the nephron, thus increasing natriuresis and kaliuresis.

## Conclusion

In this study, it is evident that the aqueous extract leaves of *V. heterophylla* have potent and dose-response diuretic and antioxidant properties in experimental animal model. In view of all these various uses associated with these compounds found in *V. heterophylla* leaves extract, we recommend further research on this plant leaves to quantify the concentration of these compounds per known amount for industrial use. We believe these compounds in *V. heterophylla* leaves could be harnessed for industrial and medicinal sciences utilization. The present study has also confirmed the ethnopharmacological use of the aqueous extract leaves of *V. heterophylla* as a diuretic agent, but further studies are necessary to evaluate the mechanisms involved in its biological activity and safety following repeated exposure. Furthermore, this work supports the importance of the preservation of local knowledge as well as the conservation of Cameroonian biodiversity

## Abbreviations

%: Percentage
CAI: Carbonic anhydrase inhibition
CAT: Catalase
CH_2_0: Free water clearance
Cosm: Osmolar clearance
CreatC: Creatinine clearance
GFR: Glomerular filtration rate
HCTZ: Hydrochlorothiazide
HNC: National Herbarium of Cameroon
LANAVET: National Veterinary Laboratory
MDA: Malondialdehyde
NO–cGMP: Nitric oxide -cyclic guanosine monophosphate
ROOH: 'Hydroperoxyde
Uosm: Urinary osmolarity
*V. heterophylla*: *Vepris heterophylla*
